# Prescription Audit as a Quality Improvement Tool: Impact of Repeated Interventions in Paediatric Outpatient Department (OPD) and Inpatient Department (IPD) Settings

**DOI:** 10.7759/cureus.98960

**Published:** 2025-12-11

**Authors:** Prajwal B Gadgeesh, Vikram Sakaleshpur Kumar, Niveditha Hegde Venkatramana, Latha S P

**Affiliations:** 1 Pediatrics and Neonatology, Subbaiah Institute of Medical Sciences, Shivamogga, IND; 2 Pediatrics and Child Health, Subbaiah Institute of Medical Sciences, Shivamogga, IND; 3 Pediatric Medicine, Sarji Maternal and Child Hospital, Shivamogga, IND; 4 Pediatrics, Subbaiah Institute of Medical Sciences, Shivamogga, IND

**Keywords:** antibiotic stewardship, lmic, low-resource hospitals, pdsa, pediatric pharmacology, prescription audit, quality improvement, who indicators

## Abstract

Background

In many low- and middle-income country (LMIC) hospitals, prescription auditing is rare due to limited digital infrastructure and weak quality-assurance systems. We evaluated whether repeated audit-feedback cycles could improve prescription completeness and rational drug use in an LMIC tertiary pediatric setting.

Methodology

Prospective, repeated cross-sectional audits (10 cycles; Nov 2023-Nov 2024) were performed in Pediatrics/Neonatology at a tertiary hospital in India. Cycles used a manual, paper-based process with structured feedback and brief prescriber education; a re-audit occurred eight months later (Plan-Do-Study-Act (PDSA) model). The primary objective of this study was to evaluate the effectiveness of a targeted audit and feedback intervention as a strategy to achieve sustained improvement in the overall quality of prescribing practices and documentation standards within our paediatric facility over consecutive PDSA cycles. IBM SPSS Statistics for Windows, Version 26 (Released 2018; IBM Corp., Armonk, New York, United States) with the Cochran-Armitage Trend Test for trend and paired *t*-tests at *p*<0.05 were performed.

Results

Documentation of OPD numbers improved substantially from 50% to 99%; legibility increased from 30% to 90%; and generic prescribing rates rose from 60% to 92%. The mean number of drugs per prescription declined significantly from 4.4 to 1.5 (p<0.01), and antibiotic use decreased markedly from 80% to 20% (p<0.01). Allergy documentation rose modestly (0%→32%). Re-audit showed small regressions (e.g., antibiotics 30%), indicating need for continuous reinforcement.

Conclusions

Even without electronic systems, cost-neutral, audit-feedback cycles in an LMIC setting produced substantial gains in prescribing quality and stewardship. Sustained improvement requires institutionalization of audits within governance structures.

## Introduction

A prescription serves as both a clinical and legal document, reflecting the quality of care and communication between physicians, pharmacists, and patients. Despite increasing awareness of rational drug use, prescription errors and incomplete documentation persist, particularly in low- and middle-income countries (LMICs). The World Health Organization (WHO) estimates that nearly half of all medicines are prescribed, dispensed, or sold inappropriately, contributing to drug resistance, adverse reactions, and higher treatment costs [1-3]. Adverse drug responses, medication resistance, and drug interactions are also brought on by these types of treatments. In the end, it raises the patient's mortality, morbidity, and financial burden. According to a research study by Bates et al. that evaluated adverse medication occurrences, 28% of them may be avoided. According to the study's findings, 56% of these avoidable adverse events happened during the prescription ordering process [4].

Prescription audits are structured quality improvement (QI) tools that evaluate adherence to evidence-based prescribing standards and identify opportunities for improvement [3,4]. Regular audits, when coupled with prescriber feedback, can markedly enhance documentation, rational drug use, and patient safety [5-8].

While numerous Indian studies have assessed prescribing patterns separately in outpatient or inpatient settings [9-20], comparative and longitudinal data from paediatric departments remain limited. This study aimed to evaluate the impact of repeated prescription audit-feedback cycles on prescribing practices in both OPD and IPD settings of a tertiary-care teaching hospital.

Unlike high-income settings with institutionalized, electronic audit systems, this work was undertaken in an LMIC country hospital with limited digital infrastructure, paper-based prescribing, and nascent quality-assurance mechanisms. Demonstrating sustained improvement under these constraints reframes prescription auditing as a feasible, scalable, and cost-neutral strategy for similar LMIC facilities. The necessity for continuous monitoring through robust frameworks like the Plan-Do-Study-Act (PDSA) cycle is evident in both local and international literature. The primary objective of this study was to evaluate the effectiveness of a targeted audit and feedback intervention as a strategy to achieve sustained improvement in key prescribing quality indicators within our paediatric facility over consecutive PDSA cycles, utilizing established World Health Organization (WHO) core prescribing indicators and India’s 2021 NHSRC audit guidelines [21].

## Materials and methods

Study design and setting

A prospective, repeated cross-sectional study was conducted over 12 months (November 2023 - November 2024) in the Departments of Pediatrics and Neonatology at Subbaiah Institute of Medical Sciences, Shivamogga, Karnataka, India. Ten audit cycles were carried out, followed by a re-audit eight months later.

Feasibility and resources

All audits were conducted using existing departmental staff, paper prescriptions, and manual tabulation. No external funding, software, or additional personnel were required; sessions were integrated into routine meetings (cost-neutral model).

Quality-improvement framework

**Figure 1:**
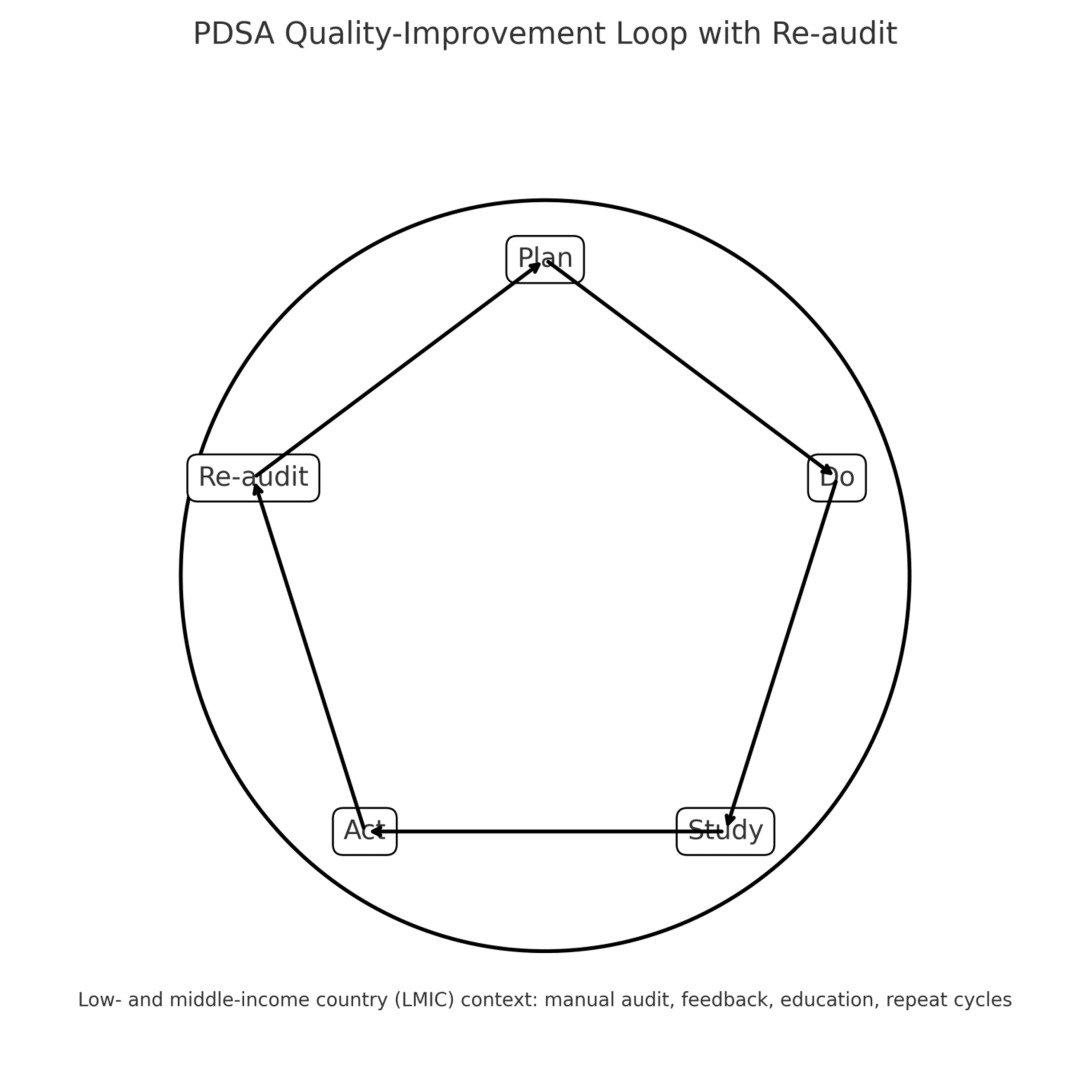
PDSA quality-improvement loop

Cycles followed a PDSA loop: Plan (define indicators, sampling), Do (collect/audit), Study (analyse/feedback), Act (education/reminders/template tweaks), and Re-audit to assess retention (Figure 1).

Study aim and measurable outcomes

The specific aim of our initial PDSA cycles was to improve overall prescription quality through targeted interventions. The primary objective was to evaluate the effectiveness of a targeted educational intervention aimed at reducing prescribing errors. To achieve this, our analysis specifically focused on a subset of data meeting the inclusion criterion of containing at least one prescribing error. This approach enabled us to track changes in error prevalence and type over time and directly measure the impact of our quality improvement initiative, rather than general prescribing patterns. Measurable outcomes were defined using several key performance indicators based on the WHO/INRUD core prescribing metrics and facility documentation standards. We aimed to optimize the following metrics through our stewardship intervention, moving from our baseline values toward established optimal ranges: the average number of medicines per prescription (Baseline 4.4; Target 2.0; Optimal 1.6-1.8), the percentage of medicines in generic names (Baseline 60%; Optimal 100%), medicines prescribed as per EML/Formulary (Baseline 100%; Optimal 100%), antibiotics prescribed (Baseline 80%; Optimal 20.0%-26.8%), and injections prescribed (Baseline 10%; Optimal <20%). Additionally, we targeted 100% compliance for key documentation parameters that showed significant improvement following feedback sessions, including complete patient name (baseline 70%), correct age and date mentioned (baseline 80% for both), clearly written dose/schedule (baseline 40%), and ensuring prescriptions were duly signed (baseline 20%). By measuring these indicators across consecutive cycles, we aimed to assess the effectiveness of the feedback mechanism on both general prescribing patterns and documentation standards.

Ethical approval

Ethical clearance was obtained from the Institutional Ethics Committee, Subbaiah Institute of Medical Sciences (Approval No. IEC-SUIMS/180/Oct-2025). The study adhered to the principles of the Declaration of Helsinki.

Sampling and inclusion criteria

Data collection involved a purposive sampling strategy, aligning with our objective to observe improvements in clinical prescribing practices after a feedback intervention. We collected a consistent sample of 100 prescriptions per cycle from Outpatient Department (OPD) and Inpatient Department (IPD) pediatric patients. The core inclusion criterion was the presence of at least one documentation or prescribing error, which allowed us to focus efficiently on the specific deficiencies we aimed to rectify and measure the impact of our QI efforts. 

The minimum required sample size was calculated using a formula provided within the established audit guidelines. This guideline employs a formula to determine a representative sample size based on the facility's average patient attendance, typically using a 95% confidence level with 10% margin of error. Our chosen sample size of 100 prescriptions per cycle exceeded the calculated minimum requirement of 73 samples, ensuring the statistical robustness of our findings.

Audit tool and indicators 

Auditing was based on the “1534 Prescription Audit Guidelines (2021)” of the National Health Systems Resource Centre (NHSRC), aligned with WHO/INRUD core prescribing indicators. Parameters included patient identifiers, diagnosis, drug name, strength, dosage, frequency, duration, legibility, allergy documentation, and prescriber signature [21].

Intervention

After each audit, structured feedback and brief educational sessions were conducted for prescribers, focusing on rational prescribing, generic drug use, and adherence to WHO standards. Audit findings were discussed during departmental meetings.

Statistical analysis

Data were compiled in Microsoft Excel and analysed using IBM SPSS Statistics for Windows, Version 26 (Released 2018; IBM Corp., Armonk, New York, United States). Categorical variables were expressed as percentages and compared using the Cochran-Armitage Trend Test. Continuous variables were analysed using t-tests. p < 0.05 was considered statistically significant.

## Results

A total of 10 sequential prescription audits were conducted between November 2023 and March 2024, following the 1534_Prescription Audit Guidelines (2021). The audits were performed at approximately monthly intervals. Each audit cycle was followed by prescriber feedback and sensitization sessions, focusing on improving completeness, rational drug use, and adherence to prescribing standards.

**Figure 2:**
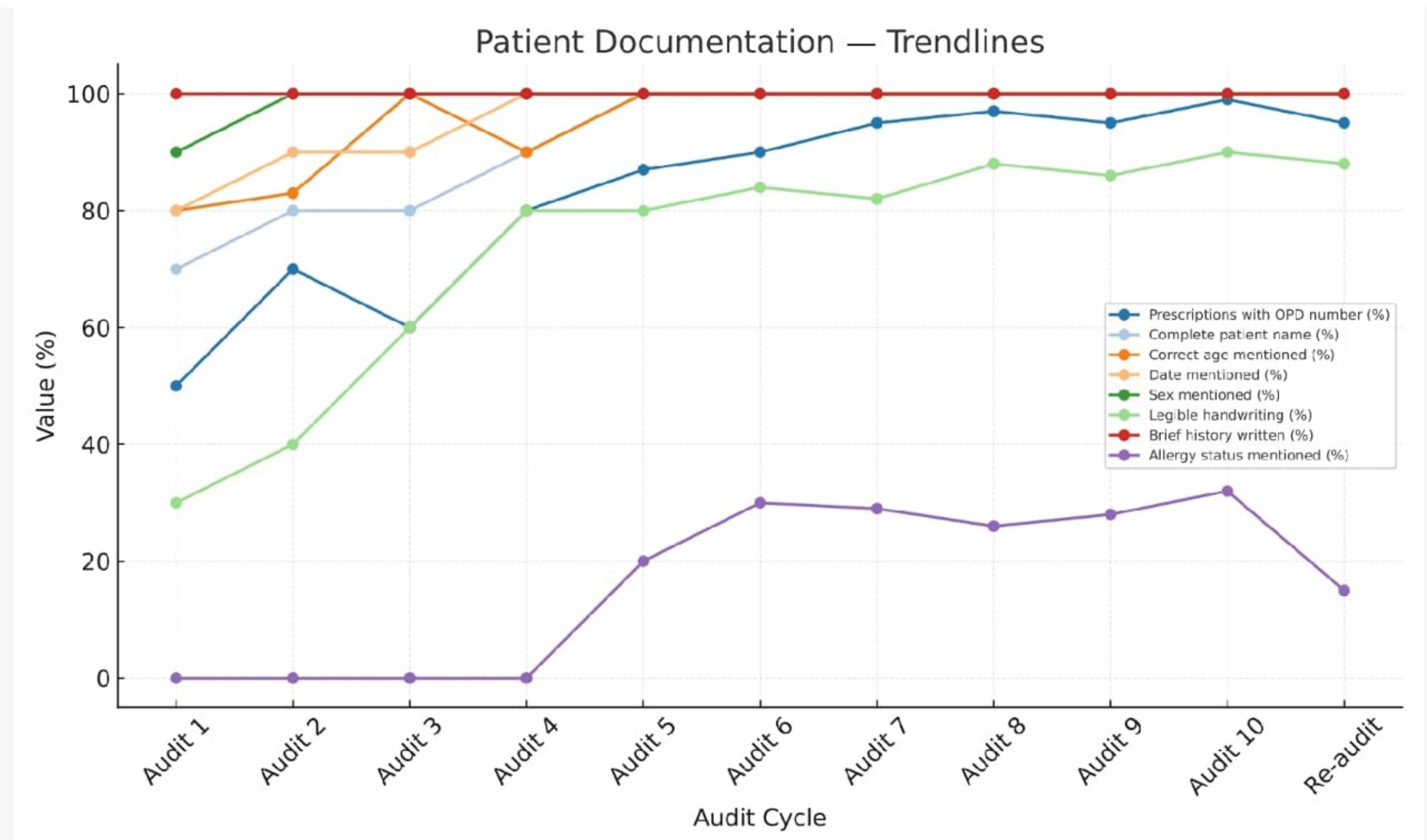
Trends in patient documentation indicators

**Figure 3:**
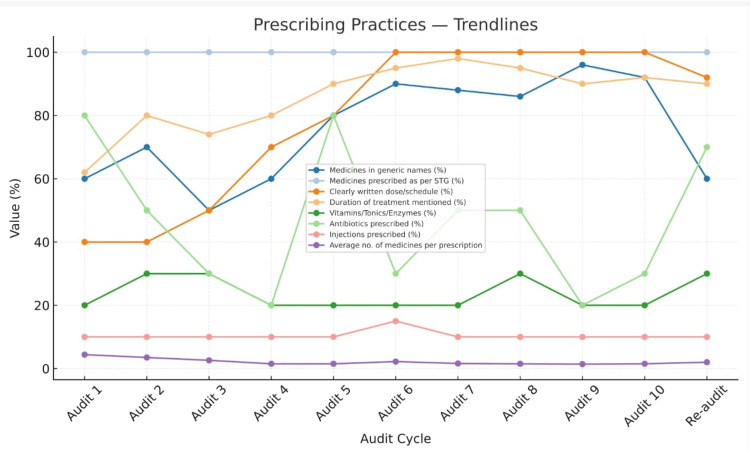
Trends in prescribing practices

**Figure 4:**
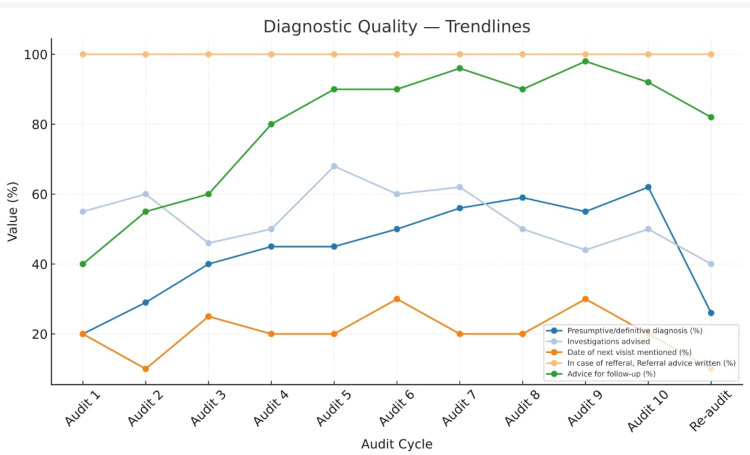
Trends in diagnostic quality indicators

Progressive improvement was observed across multiple indicators, particularly in the recording of patient identifiers, prescription legibility, diagnosis documentation, and clarity of dosage instructions, completeness of demographic data, and rational use patterns. However, allergy documentation, duration of treatment, and route of administration remained consistently low. The reduction in the average number of drugs per prescription and a transient decline in antibiotic use indicate emerging awareness toward rational drug use principles among prescribers. The rebound in antibiotic prescribing in the tenth audit suggests that continuous and sustained auditing is essential to maintain improvements over time (Figures 2-4).

Trend analysis using the Cochran-Armitage test demonstrated statistically significant improvements across several prescription quality indicators over the ten audit cycles and the re-audit. Documentation-related measures such as OPD number, patient identifiers, legibility, and clarity of dose showed significant upward trends. Prescribing indicators including follow-up advice, signature completeness, and average number of medicines also demonstrated significant changes across cycles. A subset of indicators, including allergy documentation, investigations advised, and antibiotic-related parameters, did not show statistically significant linear trends (p > 0.05). Indicators that were already near 100% compliance at baseline (e.g., clinical examination notes, STG adherence, formulary compliance, availability in dispensary) appropriately showed no statistical variation (Tables 1-3).

**Table 1:** WHO prescription indicators Data for each indicator are represented as the sample size (N) and the corresponding percentage (%), or the mean for continuous variables ("Average no. of medicines per prescription"). The table presents the calculated Z-value (test statistic) and the associated p-value obtained from the Cochran–Armitage Trend Test used to assess for a linear trend across the ordered audits and linear regression analysis for the average number of medicines per prescription. A p-value of less than 0.05 (p<0.05) was predefined as statistically significant.

Indicator	Audit 1	Audit 5	Audit 10	Z-value/t-value	p-value
Medicines in generic names (%)	60 (60%)	80 (80%)	92 (92%)	9.75	<0.01
Medicines prescribed are as per EML/Formulary (%)	100 (100%)	100 (100%)	100 (100%)	0.000	1.00000
Antibiotics prescribed (%)	80 (80%)	80 (80%)	30 (30%)	-5.76	<0.01
Antibiotics are prescribed as per the facility’s Antibiotic Policy	80 (80%)	86 (86%)	90 (90%)	2.58	0.001
Injections prescribed (%)	10 (10%)	10 (10%)	10 (10%)	0.07	0.944
Average no. of medicines per prescription	4.4	1.5	1.5	-4.72	<0.01

**Table 2:** Prescription completeness and documentation indicators Data for each indicator are represented as the sample size (N) and the corresponding percentage (%). The table presents the calculated Z-value (test statistic) and the associated p-value obtained from the Cochran–Armitage Trend Test used to assess for a linear trend across the ordered audits. A p-value of less than 0.05 (p<0.05) was predefined as statistically significant.

Indicator	Audit 1	Audit 5	Audit 10	Z-value	p-value
Prescriptions with OPD number (%)	50 (50%)	87 (87%)	99 (99%)	10.957	<0.01
Complete patient name (%)	70 (70%)	100 (100%)	100 (100%)	10.957	<0.01
Correct age mentioned (%)	80 (80%)	100 (100%)	100 (100%)	8.53	<0.01
Date mentioned (%)	80 (80%)	100 (100%)	100 (100%)	8.4	<0.01
Sex mentioned (%)	90 (90%)	100 (100%)	100 (100%)	4.32	<0.01
Legible handwriting (%)	30 (30%)	80 (80%)	90 (90%)	19.11	<0.01
Brief history written (%)	100 (100%)	100 (100%)	100 (100%)	0	1
Allergy status mentioned (%)	0 (0%)	20 (20%)	32 (32%)	1.34	0.18
Salient features of clinical examination written (%)	100 (100%)	100 (100%)	100 (100%)	0	1
Presumptive/definitive diagnosis (%)	20 (20%)	45 (45%)	62 (62%)	7.64	<0.01
Duly signed (%)	20 (20%)	95 (95%)	100 (100%)	18.01	<0.01

**Table 3:** Rational prescribing practice indicators Data for each indicator are represented as the sample size (N) and the corresponding percentage (%). The table presents the calculated Z-value (test statistic) and the associated p-value obtained from the Cochran–Armitage Trend Test used to assess for a linear trend across the ordered audits. A p-value of less than 0.05 (p<0.05) was predefined as statistically significant.

Indicator	Audit 1	Audit 5	Audit 10	Z-value	p-value
Medicines prescribed as per STG (%)	100 (100%)	100 (100%)	100 (100%)	0	1
Clearly written dose/schedule (%)	40 (40%)	80 (80%)	100 (100%)	17.53	<0.01
Duration of treatment mentioned (%)	62 (62%)	90 (90%)	92 (92%)	7.89	<0.01
Date of next visit mentioned (%)	20 (20%)	20 (20%)	20 (20%)	2.2	0.027
In case of referral, referral advice written (%)	100 (100%)	100 (100%)	100 (100%)	0	1
Advice for follow-up (%)	40 (40%)	90 (90%)	92 (92%)	13.08	<0.01
Vitamins/Tonics/Enzymes (%)	20 (20%)	20 (20%)	20(20%)	2.01	0.044
Antibiotics are prescribed as per the facility’s Antibiotic Policy	20 (20%)	20 (20%)	20 (20%)	2.58	0.001
Investigations advised	55 (55%)	68 (68%)	50 (50%)	-1.19	0.234

A comparative analysis was conducted between a baseline audit (Audit 10) and a subsequent re-audit performed eight months later to assess the sustainability of previously achieved improvements in prescribing practices and documentation quality. Data from 100 independent prescriptions were analysed for each audit. The findings reveal a mix of sustained improvement and some indicators demonstrating regression toward baseline levels without continuous reinforcement.

Compliance rates for various documentation indicators are presented in Table 4. Several foundational documentation indicators maintained optimal compliance (100%) in both audits, confirming robust adherence to basic data entry: "Complete patient name," "Correct age mentioned," "Date mentioned," "Sex mentioned," "Brief history written," and "Salient features of clinical examination written." 

**Table 4:** Comparison of prescription indicators between baseline (Audit 10) and re-audit Data for indicators are represented as percentages (%), based on a sample size of N=100 prescriptions for both audits. The statistical significance for these proportional differences was assessed using the Chi-square test. Data for indicator ("Average no. of medicines") is represented as Mean* (N=100 per audit), with statistical comparison performed using the two-sample Z-test. A p-value of less than 0.05 (p<0.05) was considered statistically significant.

Indicator	Audit 10	re-audit	x^2^/z-value	p-value		Statistically Significant (p < 0.05)
Prescriptions with OPD number	99	95	1.55	0.214		No
Complete patient name	100	100	0	1		No
Correct age mentioned	100	100	0	1		No
Date mentioned	100	100	0	1		No
Sex mentioned	100	100	0	1		No
Legible handwriting	90	88	0.051	0.82		No
Brief history written	100	100	0	1		No
Allergy status mentioned	32	15	7.12	<0.01		Yes
Salient features of clinical examination written	100	100	0	1		No
Presumptive/definitive diagnosis	62	26	24.86	<0.01		Yes
Medicines in generic names	92	60	26.34	<0.01		Yes
Medicines prescribed as per STG	100	100	0	1		No
Clearly written dose/schedule	100	92	6.38	<0.05		Yes
Duration of treatment mentioned	92	90	0.061	0.8		No
Date of next visit mentioned	20	10	3.18	0.07		no
In case of referral, Referral advice written	100	100	0	1		No
Advice for follow-up	92	82	3.58	0.058		no
Duly signed	100	86	12.98	<0.01		Yes
Medicines Prescribed are as per EML/Formulary	100	100	0	1		No
Medicines advised are available in the dispensary	100	100		1		No
Vitamins/Tonics/Enzymes	20	30	2.16		0.14	No
Antibiotics prescribed	30	70	30.42	<0.01		Yes
Antibiotics are prescribed as per the facility's Antibiotic Policy	90	86	426	0.51		No
Investigations advised	50	40	1.64	0.2		No
Injections prescribed	10	10	0	1		No
Average no. of medicines per prescription	1.5	2	-3.42	<0.01		Yes

Significant declines in compliance were observed in the re-audit compared to the baseline for several crucial items like mentioned allergy status saw a significant drop in compliance from 32% to 15% (p = <0.001), suggesting a decline in safety practice without reinforcement. Compliance decreased significantly in writing diagnosis from 62% in the baseline audit to just 26% in the re-audit (p < 0.001). The proportion of duly signed prescriptions dropped significantly from 100% at baseline to 86% in the re-audit (p < 0.001). Compliance with the mentioned next date of visit dropped from 20% to 10% (p = 0.022), indicating a significant decrease in planning for follow-up care.

Changes in prescribing quality indicators showed mixed results. A significant decrease was observed in medicines in generic names, falling from 92% to 60% (p < 0.001), indicating a strong regression in the practice of using generic names. The proportion of prescriptions including antibiotics increased significantly from 30% to 70% (p < 0.001). Interestingly, the proportion of Vitamins/Tonics/Enzymes prescribed increased significantly from 20% to 30% (p = 0.14, not significant but trending).

The average number of medicines prescribed per prescription showed a significant increase in the re-audit (Mean = 2.0±1) compared to the baseline audit (Mean = 1.5±1). This difference was highly statistically significant (Z=−3.42, p<0.01), suggesting an increase in polypharmacy over the eight-month period without intervention.

## Discussion

The findings demonstrate progressive improvement in several key prescription indicators, highlighting the effectiveness of structured audit-feedback mechanisms as a sustainable QI tool.

Improvement in prescription completeness and legibility

One of the most notable outcomes was the marked improvement in prescription completeness and legibility. Recording of patient identifiers such as OPD number, name, age, and sex reached near-total compliance by the fifth audit cycle and 100% compliance by the end of the 10th cycle (Figure 2). Similar findings were reported by Ninnekar et al. (2024) [20] and Dwivedi et al. (2024) [11], where repeated auditing significantly enhanced the recording of patient details and reduced administrative errors. Legibility, which initially was poor (30%), improved to 80% after repeated feedback, comparable to the improvement observed by Jan et al. (2024) [18] in Anantnag, where structured feedback following each audit cycle led to better handwriting clarity and standardized prescription formats.

Documentation gaps and persistent deficiencies

Despite significant progress, persistent deficiencies were noted in the documentation of allergy status, route of administration, and duration of therapy, all of which remained at 0% throughout all audit cycles in the beginning and gained some improvement in further cycles (Figures 2-4). These gaps mirror findings from earlier studies by Chandran (2024) [19] and Moideen et al. (2025) [16], who observed similar omissions even after multiple interventions. The absence of allergy documentation remains a widespread issue in Indian hospitals, often due to the lack of standardized fields in prescription formats. These results underline the need for institutional-level policy changes, such as revising prescription templates to include mandatory fields for allergy status and route of administration.

Rational drug use

The study demonstrated a substantial reduction in the average number of medicines per prescription from 4.4 in Audit 1 to 1.5 by Audit 5, and moving to 1.5 in Audit 10, indicating a clear movement toward rational prescribing. This aligns with WHO’s recommendation of an average of 1.6-1.8 drugs per encounter and reflects effective implementation of rational drug use principles through ongoing monitoring and prescriber education (Figure 3). Similar downward trends in polypharmacy were reported by Kumar et al. (2020) [12] and Solanki & Shah (2015) [9] following regular audit-feedback interventions.

Antibiotic prescribing trends

A notable observation was the fluctuation in antibiotic prescribing-declining from 80% in the first audit to 20% in the fourth, followed by a resurgence to 80% in the fifth cycle. This temporary improvement reflects the short-term success of feedback sessions, but the rebound suggests that sustained auditing and continuous reinforcement are necessary to achieve lasting behavioral change. After in further cycles showed a decline in the average number of antibiotics prescribed, reaching 30 % in audit 10 (Figure 3). Comparable patterns have been described in the literature; Parveen et al. (2024) [10] and Gupta & Rukmini (2023) [15] both noted that antibiotic stewardship gains tend to diminish over time without consistent audit reinforcement.

Impact of feedback and education

The improvements across multiple indicators following each audit cycle underscore the value of feedback-driven QI strategies. Every cycle provided an opportunity for corrective measures through prescriber sensitization sessions, reminders about generic prescribing, and reinforcement of WHO and NLEM (2022) guidelines. Moideen et al. (2025) [16] also demonstrated that regular feedback based on structured checklists could significantly improve prescription completeness and rationality within a tertiary-care setting. Similarly, Chandran (2024) [19] emphasized that prescription audits serve as an essential feedback mechanism. This project highlights how audit-feedback cycles can serve as dynamic QI tools. Periodic re-audits, visual dashboards, and prescriber scorecards can sustain motivation and ensure accountability. Integration with hospital Drug and Therapeutics Committee (DTC) activities is recommended for long-term institutionalization.

Comparison with LMIC realities and global relevance

This project shows that human-driven, low-cost QI can correct documentation gaps, reduce polypharmacy, and curb antibiotic overuse in LMIC hospitals. Gains comparable to electronically supported programs were achieved with paper-based workflows, aligning with WHO rational use targets and demonstrating transferability to district hospitals and teaching institutions without EHRs.

Feasibility, sustainability, and policy implications

The model is cost-neutral (no software/licensing; leverages existing meetings) and thus well-suited to LMIC budgetary constraints. To sustain effects and avoid regression seen on re-audit, we recommend: (i) quarterly audit cycles, (ii) simple standardized prescription templates with mandatory allergy/route/duration fields, (iii) unit-level dashboards, and (iv) Drug & Therapeutics Committee oversight. Affordable, lightweight e-prescription modules may further reduce omission errors.

Limitations

The present study has several limitations. The primary limitation is that our analysis focused exclusively on prescriptions containing at least one error. While effective for a targeted QI initiative, this approach may have inflated deficiency rates and limit the generalizability of our findings to all prescriptions within the facility. Additionally, the absence of interdepartmental stratification and the lack of clinical outcome measurements (e.g., reduction in adverse drug events) are acknowledged limitations. Specifically concerning antibiotic utilization, our analysis relied on high-level, facility-wide volume indicators (WHO/INRUD metrics). We did not conduct a granular, patient-level review linking each prescription to a specific diagnosis and local treatment guideline. Future research would benefit from multicentric audits that incorporate robust outcome metrics and leverage digital prescription platforms for more comprehensive data collection.

Recommendations 

To improve prescription practices, hospitals should institutionalize routine audits by conducting quarterly prescription reviews as part of the QI framework. Implementing standardized prescription templates with mandatory fields for allergy information, route, and duration can further enhance accuracy and completeness. Continuous prescriber education should be integrated through CME sessions and audit discussions during departmental meetings, reinforcing best practices. Transitioning to digital prescribing systems can improve legibility and incorporate automatic completeness checks, while an audit-feedback loop, displayed on departmental dashboards, promotes transparency and accountability. Linking these efforts with clinical governance, including oversight by the DTC and integration into pharmacovigilance programs, ensures alignment with institutional safety standards. Finally, future research should explore the relationship between prescription completeness and patient safety outcomes to guide ongoing improvements.

## Conclusions

Repeated prescription audits with structured feedback substantially improved documentation completeness, rational drug use, and antibiotic stewardship in pediatric OPD and IPD settings. However, sustaining these achievements requires institutionalization of regular audit cycles and integration into hospital quality governance systems.

This LMIC experience confirms that meaningful prescribing improvements can be delivered without new funding or digital systems, provided there is clinical leadership, iterative feedback, and accountability.
